# Cell size and cancer: a new solution to Peto's paradox?

**DOI:** 10.1111/eva.12228

**Published:** 2014-11-07

**Authors:** Sebastian Maciak, Pawel Michalak

**Affiliations:** 1 Virginia Bioinformatics Institute, Virginia TechBlacksburg, VA, USA; 2 Institute of Biology, University of BialystokBialystok, Poland

**Keywords:** cancer, cell size, metabolism, Peto's paradox

## Abstract

Cancer, one of the leading health concerns for humans, is by no means a human-unique malady. Accumulating evidence shows that cancer kills domestic and wild animals at a similar rate to humans and can even pose a conservation threat to certain species. Assuming that each physiologically active and proliferating cell is at risk of malignant transformation, any evolutionary increase in the number of cells (and thus body mass) will lead to a higher cancer frequency, all else being equal. However, available data fail to support the prediction that bigger animals are affected by cancer more than smaller ones. The unexpected lack of correlation between body size (and life span) and cancer risk across taxa was dubbed Peto's paradox. In this perspective, several plausible explanations of Peto's paradox are presented, with the emphasis on a largely underappreciated relation of cell size to both metabolism and cell division rates across species, which we believe are key factors underlying the paradox. We conclude that larger organisms have bigger and slowly dividing cells with lower energy turnover, all significantly reducing the risk of cancer initiation. Solving Peto's paradox will enhance our understanding the evolution of cancer and may provide new implications for cancer prevention and treatment.

## Introduction

According to the last WHO report (IARC 2012), only in 2012 about 8.2 million people worldwide did die of cancers, and currently only up to 30% tumor types can be prevented. For this reason, understanding genetic and molecular mechanisms initiating cancer and controlling its progress has been essential over decades. As most cancers develop through the accumulation of deleterious mutations, each physiologically active and proliferating cell is at risk of malignant transformation. Even though the risk is extremely low for a single cell, the probability of cancer initiation will rise with increase of both life span and body size (i.e., number of cells). Long-lived, multicellular organisms should thus have higher probability of cancer development due to increase in the number of cell divisions (and associated errors), accumulation of toxic byproducts of cell physiology (e.g., reactive oxygen species, ROS), and prolonged negative effects of external environment (e.g., viruses, bacteria, and external toxins) (Caulin and Maley 2011; Dang 2012; Nunney 2013). However, available data fail to confirm any correlation between chances of getting cancer and body mass or longevity across a broad range of species, and the absence of such a relationship has been known as Peto's paradox (Peto et al. 1975; Leroi et al. 2003; Caulin and Maley 2011; Roche et al. 2012, 2013).

Interest in Peto's paradox resurges after it has been suggested that its solution can provide new methods of cancer prevention and treatment (Bredberg 2009; Caulin and Maley 2011; Roche et al. 2013). If Peto's paradox describes a real phenomenon, natural selection has had a very important role to play in the enhancement of cancer resistance in, for example, blue whales weighing over 100 tons relative to house mice weighing 20 g or less. Thus, understanding how natural selection responds to cancer challenges in other species can be illuminating for biomedical sciences as well. Hypothetical compensatory mechanisms to be driven by natural selection may include slower somatic cell turnover, redundancy of tumor suppressor genes, more efficient immune system, better suppression of inflammation, or enhanced resistance to oncogenic viruses (Leroi et al. 2003; Roche et al. 2013). To date, evidence for changes in the mechanisms of cancer suppression between species has been scarce. However, latest insights into the cell genetics and ecological physiology can shed new light on Peto's paradox and suggest new areas for empirical testing toward its solution. Here, we outline several physiological factors influencing cancer incidence in relation to Peto's paradox, with the emphasis on cell size variation between species, a critical, albeit largely underappreciated, factor that can be a key to solving the paradox.

## Body size as an implication of cell number and cell size

There is little doubt that in general bigger animals are built of a greater number of cells. Assuming that every single cell has the same probability of getting cancerogenic mutations, the cancer incidence should increase with greater numbers of cells at risk (Calabrese and Shibata 2010). Hence, organisms built of more cells should have greater chance to develop cancer than the smaller ones. For example, Caulin and Maley (2011) estimated that if there were no additional mechanisms of tumor suppression in big animals, all whales would die of colon cancer by the age 90, in contradiction with the fact that they actually belong to the longest living animals. It simply follows from the assumed equation for probability of colon cancer initiation (*P*):

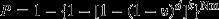


where *u* is the mutation rate, *d* is the number of stem cell divisions, *k* is the number of rate limiting mutations required for cancer to occur, *N* is the number of effective stem cells, and *m* is the number of crypts in colon (cell number) [for more details see Calabrese and Shibata 2010; see also Nunney (2013) for another model]. According to Caulin and Maley's (2011) estimation, the probability of getting colon cancer in whales at the age of 90 equals 1, which means 100% chance for cancer development in every individual.

However, it is worth noticing that the increase in organismal body size is not a function of cell quantity alone (isometric scaling) but a combination of both cell number and cell size (Kozłowski et al. 2003, 2010). Also, there is no reason to expect that the cell division rate is constant across species. A strong positive correlation between body size and cell size at inter- and intraspecific levels was found in many groups of vertebrates, throughout a broad range of mass and cell sizes (Kozłowski et al. 2010; Maciak et al. 2011a; Starostová et al. 2013). For example, mammalian erythrocytes vary from ∼78 *μ*m^2^ to ∼215 *μ*m^2^ in pygmy shrew and elephant, respectively (Gregory 2004). Therefore, all attempts to resolve Peto's paradox should take into account not only the cell numbers but also changes in cell size.

An increase in cell size leads to many morphological and physiological consequences including those directly related to cancer. One of them is the observed negative correlation between cell size and cell division rate (Gregory 2001, 2002, 2005; Neumann and Nurse 2007), which due to possible errors during each genome copy can lead to a malignant transformation. Any evolutionary enlargement of body size should then occur as a consequence of increases in cell number, as well as an increase in size of slower dividing cells. The size of mature cells results from an evolutionary trade-off between growth rate and their division rate (Gregory 2002; Wells 2002; Jorgensen and Tyers 2004; Savage-Dunn 2008; Maciak et al. 2014). The trigger between these two cellular states is regulated by growth or nutritional factors, through associated specific secondary mediators in a so-called growth-signaling network (Saucedo and Edgar 2002; Marion et al. 2004; Guertin and Sabatini 2005). Because of the complexity of the process, data related to cell division rates in different organisms are scarce. In mice, colon crypt cell division rate was estimated as once per day using stem cell marker Lgr5 (Barker et al. 2007). Although to our knowledge no such data exist for humans or whales, we can fairly assume that the size of human and whale colon crypts are bigger than those in mice, and thus their cell division rate should be considerably lower (the size of whale erythrocytes is almost twice as big as in mice, with area of ∼110, ∼170, and ∼215 *μ*m^2^ in mice, human, and whales, respectively, Gregory 2004; taking erythrocyte size as a proxy for other cell sizes is justified by strong correlations of cell sizes across various tissues, Kozłowski et al. 2010; Maciak et al. 2014).

**Figure 1:**
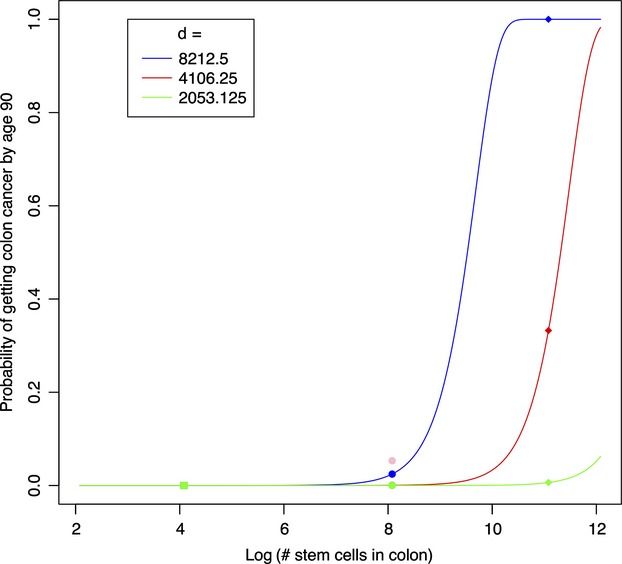
Estimated probability of colon cancer development in mouse (square), human (circle), and whale (diamond) characterized by different number of stem colon cells and the same number of stem cells divisions (d) for each animal (blue line). Red and green line indicate this estimation with number of stem cells divisions lowered by two and four, respectively.

When we incorporate different numbers of cell divisions (*d*) into the Caulin and Maley's (2011) original estimation, the probability of cancer development (*P*) in big animals significantly decreases (Fig.1). Assuming that whale's crypt cells division rate is at least twice as low as in human, estimated probability of colon cancer development in the former falls into the range observed in both mice and humans (Fig.1). Our estimates agree well with original calculations by Calabrese and Shibata (2010) who showed that a 10% increase in stem cell division rate should increase cancer risk by 1.8-fold and vice versa—lower division rate decreases risk of tumor development (see, Fig. 3 in Calabrese and Shibata 2010). The result calls in question this and other proposed models (e.g., Nunney 2013; Roche et al. 2013) that do not take into account cell sizes and associated changes in the division rate which alone can be sufficient to solve Peto's paradox. However, there are yet a couple of other plausible explanations of why big animals, despite having a greater cell number, can still have a similar chance of developing cancer to that in small ones.

## Metabolic rate as a main cause of cell condition

**Figure 2:**
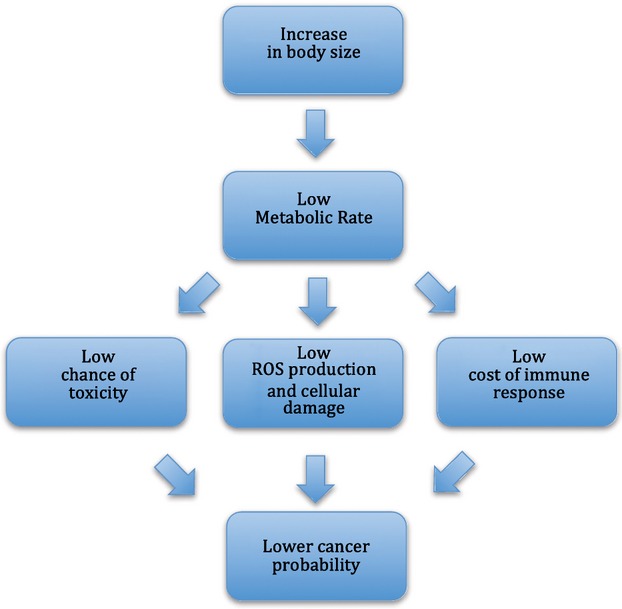
Scheme presenting metabolism-related pathways of cancer probability in big animals in accordance to Peto's paradox.

Apart from the number of cell divisions and its role in the mutation rate, metabolism is one of the most important factors influencing cancer development in many ways (Fig.2) (see, Dang 2012 for a review). For example, internal energy turnover controls the rate of cellular organelles exhaust, production of ROS, effectiveness of cellular defense systems, as well as ability to remove toxins, and mutagens from the cellular matrix.

It has been well-known that at the intraspecies level organismal metabolism rate scales allometricaly to the body mass with the exponent ranging from 0.66 to 1 (Kozłowski et al. 2003; McNab 2008; White 2010). In other words, bigger individuals have lower metabolic rate per unit of body mass than smaller individuals. Additionally, a strong negative relationship between the metabolic rate and cell size was found across a broad range of taxa (Kozłowski et al. 2003, 2010; Starostová et al. 2009; Maciak et al. 2011a; Zhang et al. 2014). Hence, evolutionary coupling between cell size and body mass has been postulated to play an important role in shaping the mass scaling of metabolism (Davison 1955; Kozłowski et al. 2003, 2010; Maciak et al. 2014).

### Cells ageing and malfunctions as a consequence of metabolic rate

Most of O_2_-mediated processes occur in mitochondria or cellular matrix (Alberts et al. 2002). Release of ROS and oxidization of biological membranes, proteins, nucleic acids, and related compounds lead to dysfunction of the biomolecules, cell ageing, and carcinogenesis (Rattan 2006; Valko et al. 2007; Dang 2012). In normal conditions, ROS are decomposed by cellular enzymes (dismutase, catalases, and peroxidases) to oxygen and water, but increased concentration of free radicals results in oxidative stress and cellular damage. As the rate of ROS production in a cell is a function of basal metabolic rate (BMR), organisms characterized by high BMR are subject to an increased risk of protein structure alterations, DNA mutations, and cancer (Ku et al. 1993; Caulin and Maley 2011). The high-energy turnover is also associated with lipid membrane peroxidation, negatively affecting multiple cellular functions (Sohal et al. 1984, 2002; Rahman 2007). Lower metabolic rate should in turn decrease cellular damage, mutation rate, and hence the risk of tumor development. For example, a dietary-restricted decrease in BMR presumably improves the resistance to oxidative stress through decreased unsaturation index in cell membrane lipids (Merry 2002; Hulbert 2005; Hulbert et al. 2007). Although the relationship between the rate of metabolism and the rate of cellular damage is well accepted, the influence of metabolism on overall life span remains controversial. The ‘rate of living – free-radical damage’ theory (Harman 1956; Sohal et al. 2002) suggests that higher rates of energy turnover should be negatively linked to life span, as it is with organelle exhaust, while the ‘uncoupling to survive’ hypothesis (Brand 2000; Speakman 2005) suggests that the correlation is positive. However, there is little doubt that a kind of evolutionary trade-off between the rates of metabolism and the ability to sustain effective defense mechanisms exists, rewarding big animals for their lower basal metabolism.

### Immune responses as an integral part of the energy budget

Another possible consequence of higher metabolic rate is compromised immunity. The ability of every multicellular organism to fight off dysfunctional, mutated cells (tumors), and/or external factors that can lead to the mutations constitutes an important mechanism controlled by natural selection. According to the life-history theory, any considerable increase in energy expenditures for processes contributing to the animal's fitness (physiological maintenance, reproduction or survival) can compromise immune responses (Stearns 1992). For example, high metabolic costs related to increase in parental investments, thermoregulation, or predatory pressure significantly decrease the effectiveness of immune responses (Cichoń et al. 2001, 2002; Książek et al. 2003). It has also been observed that animals with heritable high BMR respond worse to the immunological stress than their cousins with lower BMR (Książek et al. 2003; Książek and Konarzewski 2012). Predictably, immunodeficient mice develop more carcinogen-induced and spontaneous cancers than wild-type mice, and tumor cells from immunodeficient mice are more immunogenic than those from immunocompetent mice (Koebel et al. 2007). This implies that organisms with high metabolic rates are at greater risk of immune deficiency as well as of cancer development.

### Toxicity, metabolism and cancerogenesis

Influence of toxins, heavy metals, and other mutagenic factors on cellular DNA is considered a main cause of tumor development (Voth and Ballard 2005; Khlifi and Hamza-Chaffai 2010; Włostowski et al. 2010). Chronic exposure to any of these produces damage primarily to the metabolically active tissues (e.g., liver, kidneys, and intestines), including cellular degeneration and apoptosis, DNA mutations, interstitial inflammation, and whole organ dysfunction (e.g., Maciak et al. 2011b; Salińska et al. 2012). Although epidemiological, cell culture, and animal experimental studies have shown an increased cancer incidence associated with heavy metals intoxication (Sunderman 1984; Trott et al. 1995; Oller et al. 1997; Salnikow and Zhitkovich 2008), the mechanisms of carcinogenesis remain poorly understood. However, some evidence suggests that those mechanisms include metabolically related processes, such as increase in ROS production, enhancement of cytotoxicity, and genotoxicity of DNA damaging agents through inhibition of DNA repair, as well as epigenetic silencing of tumor suppressor genes (Salnikow and Zhitkovich 2008; Khlifi and Hamza-Chaffai 2010). For example, in human benzo[a]pyrene (one of the most common pollutants) -induced colon carcinoma, changes in most of the cellular pathways are observed (Donauer et al. 2012). Those responsible for generating toxic metabolites, such as the highly reactive electrophilic genotoxin and ultimal carcinogen B[a]P-7,8-dihydrodiol-9,10-epoxide which bind to nucleophilic macromolecules, including proteins and DNA, and cause mutations (Rubin 2001; Donauer et al. 2012). In mice, chronic cadmium (Cd) exposure leads to suppression of Ube2d gene (member of the ubiquitin-conjugating enzyme) expression and p53-dependent apoptosis of renal tubular cells (Tokumoto et al. 2011). Moreover, Maciak et al. (2011b) showed that Cd-induced cellular toxicity in metabolically active organs strongly depends on their metabolic rates. Mice artificially selected for high basal metabolic rate accumulated two times more cadmium in the liver, kidneys, and duodenum compared to mice with lower BMR (Maciak et al. 2011b). Additionally, Cd accumulation in this study was positively associated with iron concentration—a metabolically important element (Maciak et al. 2011b). Both Cd and Fe use the same DMT1 transporter in apical membrane of enterocytes for cellular uptake (Tallkvist et al. 2001; Ryu et al. 2004; Min et al. 2008). Although the expression of DMT1 in high BMR mice was not measured, the strong positive correlation between Fe and Cd accumulation suggests that high metabolic rate contributes to increased risk of cellular toxicity and its consequences, including cancer.

### Metabolic rate diversity

The level of energy turnover in every living cell defines the rate of its exhaustion, malfunction, and tumor incidence. Evolutionary increase in the body size itself, which is typically accompanied by lowered metabolic rate and increased size of cells, most likely reduces the chance of cancer development in big organisms (Fig.2). However, it should be noted that beside body size there are at least several other physiological and ecological factors directly affecting metabolic rates (see Starostová et al. 2009; Maciak et al. 2011a). This may explain why there are many exceptions from the allometry of metabolism, such as naked mole rats (*Heterocephalus glaber*), relatively small (∼30–35 g) rodents. Naked mole rats are characterized by much lower mass-specific BMR and longer life span (20–30 years) than expected by their size (de Magalhães and Costa 2009), as well as simultaneous resistance to both congenital and experimentally induced cancerogenesis (Edrey et al. 2011; Manov et al. 2013). Although the cell size of mole rats remains undescribed, their cells exhibit upregulation of cyclin-dependent kinase (Cdk) inhibitor, p16, which prevents cell division and favors cell growth (Sedivy 2009; Seluanov et al. 2009). The overexpression of p16 in naked mole rats, in conjunction with Cdk's inhibitor p27, seems to create a double barrier to cell proliferation and cancerogenesis.

## Conclusions

Peto's paradox does not seem to apply to intraspecies comparisons in which there are good examples of a positive correlation between body size and cancer incidence, including studies showing increased cancer risk in tall women (Green et al. 2011; Collaborative Group on Epidemiological Studies of Ovarian Cancer 2012). Similarly, large dog breeds that have lower metabolic rates exhibit higher incidence of osteosarcomas (Dobson 2013). However, we note that many dog breeds have been subject to a strong pressure of artificial selection that, beside increased tumor incidence, resulted in many other health-related disadvantages, including heart dysfunction, hip dysplasia, arthritis, and bone debilitating (Ettinger and Feldman 1995). In the case of humans, an above average height or obesity are often associated with abnormal levels of hormones and growth-related biomolecules leading to increased cell division rates (Leroi et al. 2003). The latter, in turn, result in higher cancer risk (e.g., Jenkins and Besser 2001).

In conclusion, we believe that although Peto's paradox is likely a multifactor phenomenon, its solution will remain elusive unless cell size and metabolic rate are taken into consideration. Natural selection has elaborated effective and fine-tuned tumor-suppressive mechanisms that ensure homeostasis between cancer incidence and mechanisms of resistance against it (Bredberg 2012; Kang and Michalak 2014). Anticancer factors are presumably subject to evolutionary trade-offs and further investment in anticancer defense, especially in postreproductive stages, can actually be selectively disadvantageous (Bredberg 2012). Solving Peto's paradox will be central to our understanding the evolutionary trade-offs and their relationship to metabolic parameters and underlying genetics.
